# Virtual Compound Screening for Discovery of Dopamine D1 Receptor Biased Allosteric Modulators

**DOI:** 10.1021/acs.jcim.5c00972

**Published:** 2025-09-11

**Authors:** Yang Zhou, William C. Wetsel, Steven H. Olson, Lawrence S. Barak

**Affiliations:** † Department of Cell Biology, 12277 Duke University Medical Center, Durham, North Carolina 27710, United States; ‡ Departments of Psychiatry and Behavioral Sciences, Cell Biology, and Neurobiology, Duke University Medical Center, Durham, North Carolina 27710, United States; § Mouse Behavioral and Neuroendocrine Analysis Core Facility, Duke University Medical Center, Durham, North Carolina 27710, United States; ∥ Conrad Prebys Center for Chemical Genomics at Sanford Burnham Prebys Medical Discovery Institute, La Jolla, California 92037, United States

## Abstract

The dopamine D1 receptor (D1R) is a therapeutic target for a variety of central nervous system disorders including Parkinson’s disease (PD). Challenges thus arise in the development of safer D1R therapies in limiting off-target drug activity. This issue is particularly relevant to PD therapy, where L-DOPA has been the “gold standard” drug for decades despite a problematic side-effect profile. Recent studies of G-protein and β-arrestin functionally selective signaling offer new strategies for developing superior D1R orthosteric and allosteric compounds with fewer side effects. We designed a desktop-computer drug-screening platform to examine large virtual chemical libraries for allosteric compounds binding D1R intracellular loop 2 (ICL2) determinants. Two structurally distinct hits were strong enhancers of dopamine-induced β-arrestin recruitment and inhibitors of dopamine-induced G-protein activation. The lead candidate DUSBI-A3 was highly selective for D1R over closely related dopamine receptors when assessed by β-arrestin activation, providing proof-of-concept for pursuing D1R selective, biased compounds in the treatment of PD.

## Graphical abstract

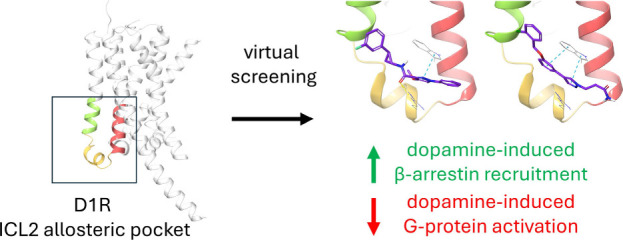

## Introduction

The dopamine 1 receptor (D1R) belongs to the 5-member G protein coupled receptor (GPCR) family whose signaling is defined by the neurotransmitter dopamine. D1R activity in dopaminergic central nervous system pathways within the striatum and prefrontal cortex plays an essential regulatory role in locomotion,^1^ reward processing,^2^ temporal control,^3^ learning,^4^ and memory.^5^ As a consequence of its critical role in dopamine physiology, the D1R has been implicated in treating a range of neurological disorders including, schizophrenia, attention-deficit hyperactivity disorder (ADHD), cognitive impairment, and Parkinson’s disease (PD).^6–9^ Due to the negative side effect profile of L-DOPA, the gold-standard therapy for PD, there is currently a considerable interest in finding less toxic D1R drugs that can supplement, postpone, or replace L-DOPA pharmacotherapy.^10^

Drug discovery efforts focused on D1R, however, face considerable practical challenges. Major hurdles include the high sequence and structural homology between D1R and other aminergic receptors that can produce serious side-effects, which complicates development of D1R-selective orthosteric ligands.^11^ Some FDA-approved D1R agonists, such as L-DOPA (which is converted to dopamine), apomorphine, and rotigotine, exemplify this problem. They are well characterized for their interactions with other aminergic receptors,^12,13^ and for example their D2R-mediated side-effect profiles include hallucinations, delusions, and illusions.^14,15^ Also of consequence are many antipsychotic drugs serving as D2R antagonists (e.g., ziprasidone and paliperidone) that possess D1R antagonism with Parkinsonism side effects.^12,16,17^ For these reasons alone, drug discovery strategies that emphasize D1R selectivity have become a high priority for designing next-generation PD therapies.

Like many GPCRs, the D1R signals through G proteins, predominantly the Gs, second messenger cAMP pathway,^10^ and β-arrestins that are scaffolding proteins for various signal transduction molecules, as well as being intimately involved in the D1R trafficking pathway.^10,18–20^ Recent studies suggest that G proteins and β-arrestins drive separate physiological responses, creating an opportunity for developing signaling-biased functionally selective ligands, that in principle will have greatly improved side-effect profiles.^21–25^ Regulation of the G protein pathway has been the mainstay of PD therapy; in particular it mediates the recovery in locomotion as suggested by the use of G protein biased ligand in animal studies,^18,26,27^ while overactivation of G protein pathway is associated with dyskinesia.^28–30^ Since activation of the β-arrestin pathway in PD improves locomotion recovery while reducing dyskinesia,^24,31^ a β-arrestin-biased D1R agonist could provide therapeutic benefits. Unfortunately, to date, there are currently no available β-arrestin-biased D1R agonists identified for this purpose.

Alternatively, allosteric ligands represent another promising approach for enhancing selectivity and signaling bias at GPCRs.^21,25^ Several D1R positive allosteric modulators are known^10,32–34^ and they interact at three distinct D1R sites.^35^ Two sites are unidentified, while the third site has been localized to a pocket formed by transmembrane domain 3 (TM3), intracellular loop 2 (ICL2), and TM4 (will be referred to as the ICL2 allosteric pocket).^34,36^ Some ligands targeting the ICL2 allosteric pocket show strong D1R selectivity over other aminergic receptors, and achieve D1R selectivity over the highly homologous D5R.^32–34^ Among these ligands is LY3154207, a new drug currently in clinical trials for PD.^10^ A functionally selective analog of LY3154207, DETQ, was identified that is biased for G protein-mediated cAMP signaling over that by β-arrestin.^33^

Currently, virtual screening has been demonstrated as an effective methodology to identify novel chemotypes for GPCRs, with several studies successfully identifying novel biased ligands acting at the orthosteric pocket of the dopamine D4 receptor.^37,38^ Nevertheless, it is unclear whether virtual screening at an allosteric GPCR site can also identify novel biased allosteric ligands. In this study, we performed a virtual screening of the D1R ICL2 allosteric pocket that identified two classes of novel allosteric ligands.

## Results

### Selection, Validation, and Analysis of the D1R Receptor Model

Four cryo-EM structures of the D1R bound to the positive allosteric modulator LY3154207 (PDB entries 7LJC,^39^ 7LJD,^39^ 7CKZ,^40^ and 7X2F^41^) are publicly available. Three structures report similar LY3154207 binding poses at the allosteric site, whereas the 7CKZ structure reported a markedly different binding pose for the modulator in the ICL2 allosteric pocket (Figure 1A). To determine a more probable structure to be used for virtual screening, we docked five compounds (Figure 1B) previously reported to bind that same allosteric pocket^10^ to two distinct models (PDB: 7CKZ and 7LJC). The docking score cohort (Figure 1C, table) indicates that 7LJC is the preferred model because it better captures in totality the critical interactions between the ligands and the D1R. Additionally, a score-in-place docking analysis that assessed the interaction strength of LY3154207 for each structure (Figure 1C) showed the 7CKZ model had a weaker set of interactions (docking score, −1.953) than the 7LJC model (docking score, −5.798). Therefore, we selected structure 7LJC as the basis for subsequent modeling and virtual screening determinations.

**Figure 1:**
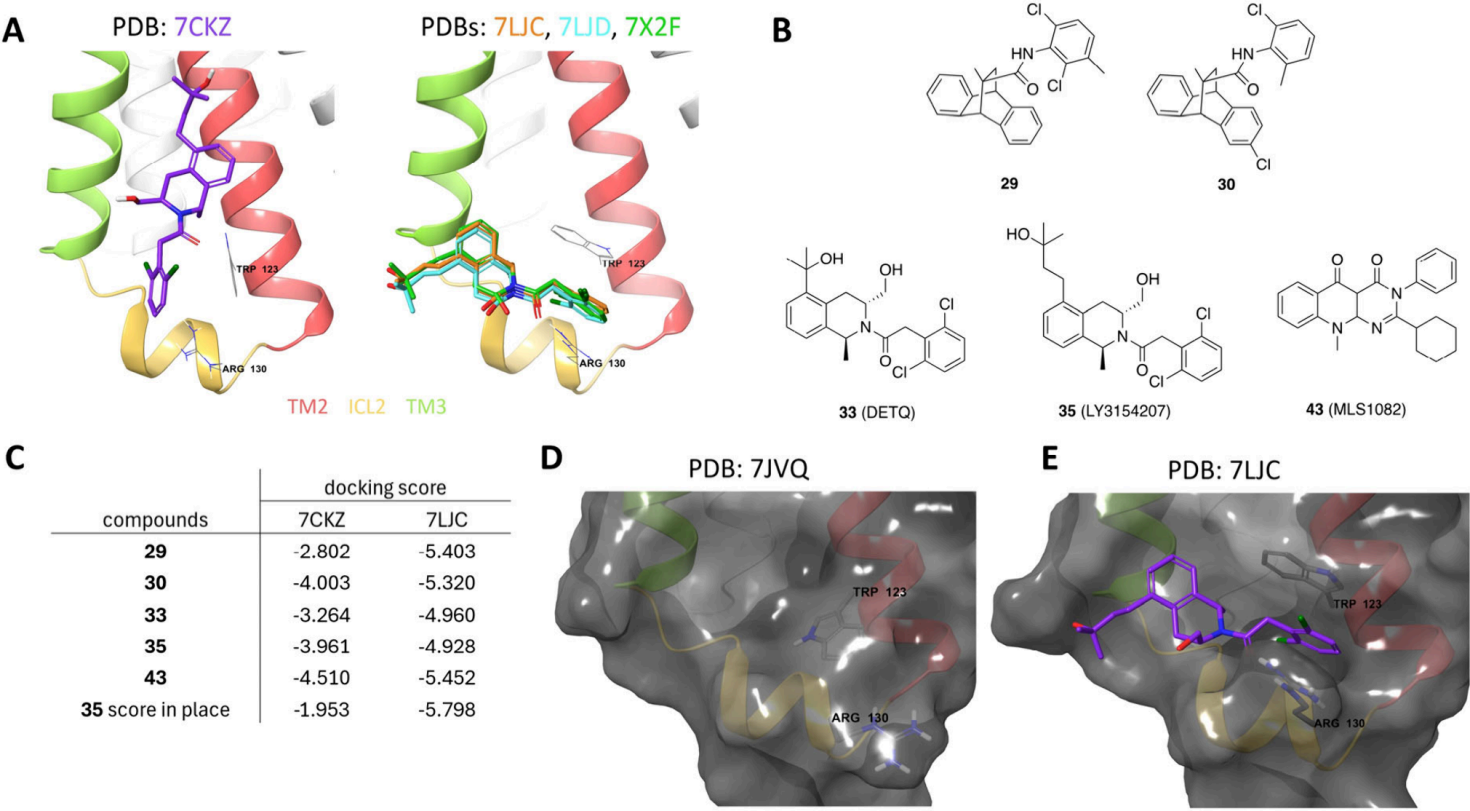
Selection, validation, and analysis of the D1R receptor model. (A) Comparison of LY3154207 ligand binding poses at the ICL2 allosteric site in PDB structures 7CKZ (left) and 7LJC (right). Depicted in the ribbon plots are TM2 (red), ICL2 (yellow), TM3 (green), and ligand LY3154207 (violet). (B) Chemical structures of known allosteric ligands that bind the human D1R (hD1R) ICL2 allosteric site. (C) Docking scores for allosteric ligands in B with hD1R structures PDB 7CKZ and PDB 7LJC. (D, E) Structural comparison of the ICL2 allosteric pocket in hD1R in the absence (PDB: 7JVQ) and presence (PDB: 7LJC) of the allosteric ligand LY3154207. The receptor electronic surface is shown in gray, with TM2, ICL2, TM3, and LY3154207 shaded in red, yellow, green, and violet, respectively.

To further study LY3154207 binding changes in the allosteric pocket, we compared the 7LJC structure to a D1R structure without the bound allosteric ligand (7JVQ). We found in the absence of ligand, the residues Trp123 and Arg130 adopt a configuration that does not offer a well-defined binding pocket (Figure 1D). With LY3154207 binding, however, these residues reorient to form a “channel”, stabilizing a pocket occupied by the LY3154207 benzene ring (Figure 1E). This comparison illustrates that key structural determinants can undergo significant movement during the formation of a ligand-stabilized pocket, emphasizing the importance of a prebound structure when modeling allosteric interactions.

### Virtual Screening to Identify Novel Allosteric Ligands at the Human D1R

We conducted a virtual screening targeting the allosteric pocket of the human dopamine D1 receptor (hD1R) with 7LJC as the model (Figure 2). We applied filters to 14 million compounds of the ZINC compound database to select 2.5 million candidates with a molecular weight between 350 and 500 and a log *P* value between 3 and 5. We then performed a random sampling to select 500000 compounds for further analysis that addressed redundancies in scaffold structures and limitations in compound procurement. The initial stage of screening was conducted using the high-throughput virtual screening (HTVS) mode, resulting in around 11000 compounds with docking scores better than −5.1. These compounds were then subjected to a more refined screening under the standard precision mode, yielding docking scores comparable to those from model validations. A total of 2011 virtual hits achieved docking scores better than −5.6. To ensure diversity, we performed compound clustering using a Tanimoto coefficient cutoff of 0.3, resulting in 200 unique chemical scaffolds. The entire screening process was performed on a single core of Intel i7–3770k CPU, requiring 309515 s, or approximately 3 days and 14 h.

**Figure 2:**
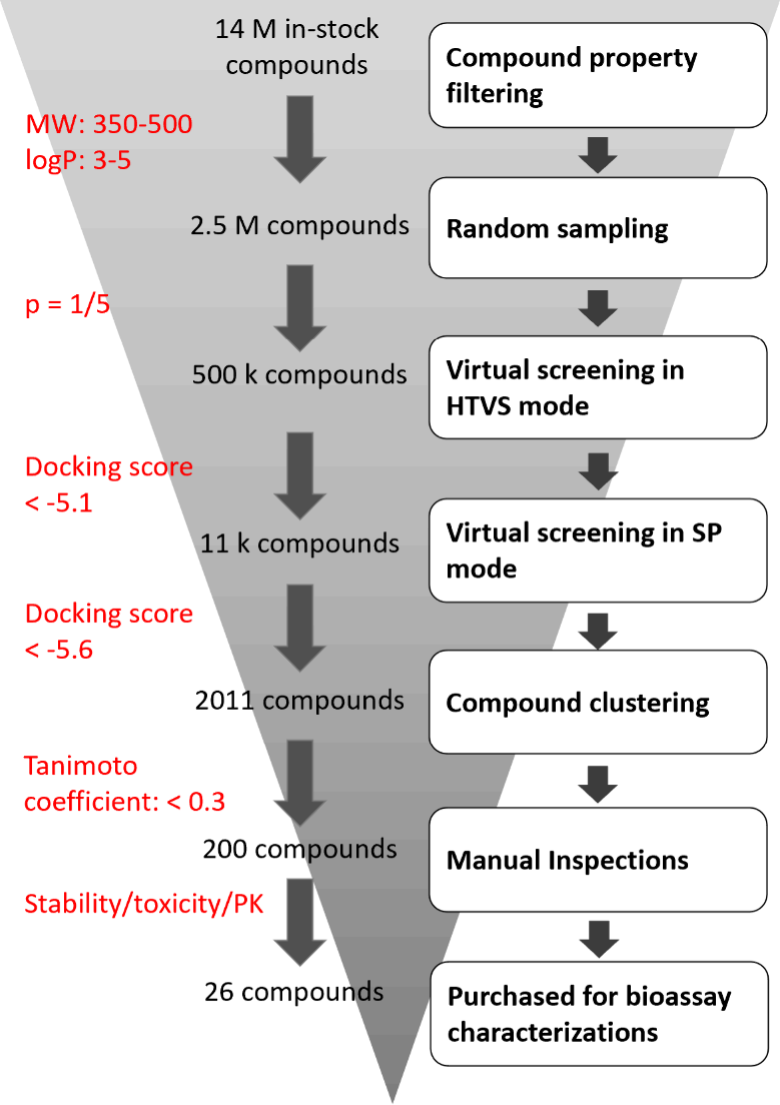
Flowchart of virtual screening at the ICL2 allosteric pocket of the hD1R.

All virtual screening hits were predicted to occupy the space between Trp123 and Arg130, likely engaging in π–π stacking interactions with Trp123 via an aromatic moiety. Some ligands were predicted to extend toward TM3, while others displayed diverse binding modes, including forming hydrogen bonds or ionic interactions with Lys134, or featuring an aromatic ring nestled in a hydrophobic pocket near TM4. After the chemical stability and potential toxicity of these scaffolds were evaluated, 26 compounds were purchased for biological evaluation.

### Discovery of Novel Arrestin-Biased Allosteric Modulators at the hD1R

We evaluated the 26 compounds using functional assays for their ability to modulate both the cAMP and β-arrestin pathways downstream of D1R with and without dopamine present. Two compounds, DUSBI-C5 and DUSBI-A3, emerged as biased allosteric modulators (Figure 3). To ensure compound purity, chirality, and reproducibility, we resynthesized these two compounds, achieving single enantiomer forms with over 95% purity. Both compounds were predicted to engage in π–π stacking interactions with Trp123 just like the reference compound LY3154207, whereas DUSBI-A3 was predicted to form a hydrogen bond with Ser127 (Figure 3A,D,G). Unlike LY3154207, which enhanced dopamine induced G protein and β-arrestin pathways through affinity shifts (Figure 3B,C), these compounds enhanced dopamine-induced β-arrestin recruitment (Figure 3F,I) while simultaneously inhibiting dopamine-induced cAMP accumulation (Figure 3E,H). Both compounds were found to strongly affect the constitutive activity and the maximum efficacy of the signaling pathways (Table 1). Compound DUSBI-C5 at 100 μM limited dopamine-induced cAMP accumulation to 45%, while it enhanced the dopamine-induced arrestin response by 90%. Compound DUSBI-A3 at 100 μM limited the dopamine-induced cAMP accumulation to 53%, but enhanced the dopamine induced arrestin response by 49%.

**Figure 3:**
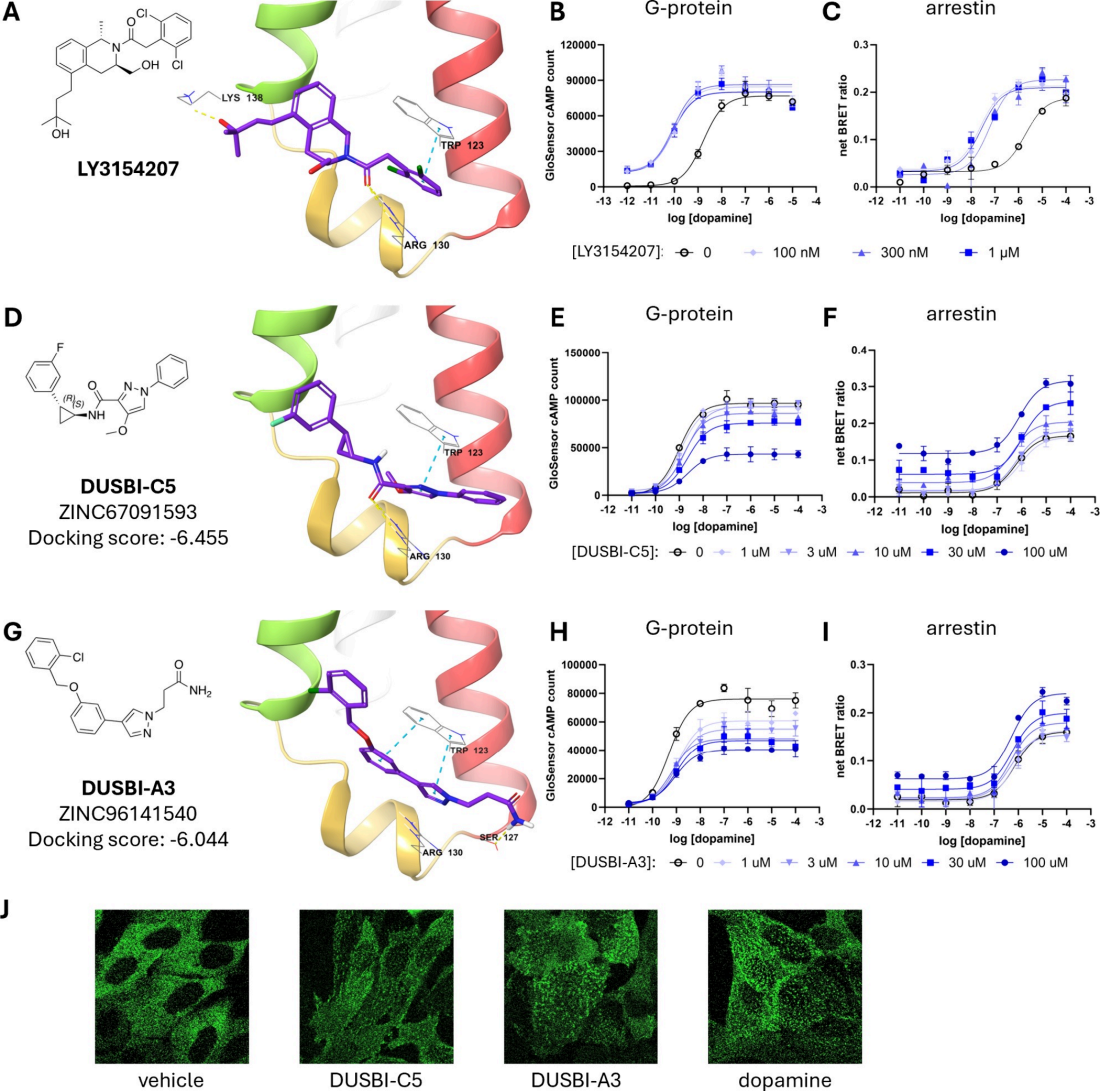
Structure and signaling of novel β-arrestin-biased allosteric modulators. (A) Structure of LY3154207. (B, C) Dose–response curves for dopamine activating the cAMP pathway (B) or the β-arrestin pathway (C) in the absence or presence of LY3154207. (D) Structure and docking pose of DUSBI-C5. (E, F) Dose–response curves for dopamine activating the cAMP pathway (E) or the β-arrestin pathway (F) in the absence or presence of DUSBI-C5. (G) Structure and docking pose of DUSBI-A3. (H, I) Dose–response curves for dopamine activating the cAMP pathway (H) or the β-arrestin pathway (I) in the absence or presence of DUSBI-A3 at different concentrations. (J) β-arrestin mediated hD1R receptor translocation with vehicle, DUSBI-C5 (100 μM), DUSBI-A3 (100 μM), or dopamine (10 μM).

**Table 1:** Best-Fit Parameters for Shifts of the Dopamine Dose-Response Curve in the Presence of Biased Allosteric Modulators (Figure 3)­^a^

	DUSBI-A3						DUSBI-C5					
	G protein			arrestin			G protein			arrestin		
[allosteric drug] (μM)	α	β	E(0)	α	β	E(0)	α	β	E(0)	α	β	E(0)
100	0.51	0.54	0.04	1.35	1.49	0.39	0.47	0.44	0.02	0.71	1.90	0.71
30	0.48	0.63	0.03	1.24	1.24	0.25	0.43	0.79	0.03	0.52	1.57	0.37
10	0.74	0.64	0.02	1.43	1.11	0.14	0.63	0.89	0.02	1.33	1.23	0.23
3	0.46	0.73	0.03	1.58	0.95	0.13	0.5	0.95	0.05	0.92	1.08	0.10
1	0.40	0.81	0.04	1.07	1.01	0.10	0.92	0.96	0.00	0.64	0.98	0.11
0	1.00	1.00	0.00	1.00	1.00	0.12	1.00	1.00	0.01	1.00	1.00	0.05

a α represents affinity shifts. β represents maximum efficacy shifts. E(0) represents constitutive activity.^25,35^ .

We derived IC_50_ values for DUSBI-C5 (31 ± 23 μM; Figure 3E) and DUSBI-A3 (5.2 ± 4.6 μM; Figure 3H) from the suppression of cAMP production at high dopamine concentrations, as reflected by reduced curve plateaus and consistent with dose-dependent inhibition. In contrast, both compounds induced β-arrestin translocation to the receptor in a dopamine-independent, dose-dependent manner, indicative of constitutive activation and reflected by elevated baseline signals at zero dopamine. Activation EC_50_ values were 30 ± 8 μM for DUSBI-C5 (Figure 3F) and 27 ± 8 μM for DUSBI-A3 (Figure 3I). DUSBI-A3 elicited a modestly stronger arrestin response than did DUSBI-C5 (Figure 3J). Collectively, these findings identify DUSBI-C5 and DUSBI-A3 as novel β-arrestin-biased allosteric modulators of D1R.

### Selectivity of the β-Arrestin-Biased Hit DUSBI-A3 to the hD1R

To evaluate the sequence homology of the ICL2 allosteric pocket, we first conducted a structural analysis, identifying 10 residues within the ICL2 allosteric site of hD1R potentially involved in ligand interactions (Figure 4A). We then performed a sequence alignment of these residues across several receptors closely related to hD1R (Figure 4B). Our findings reveal that aside from Tyr131^34.53^, which is strictly conserved among all tested receptors, the remaining residues show relatively low levels of sequence homology. Significant diversity was observed in Trp123^3.52^, Arg130^34.52^, Lys134^34.56^, and Met135^34.57^, which are key residues that were shown to have strong interactions with our virtual screening hits. These results suggest that allosteric ligands targeting the ICL2 allosteric pocket may be able to achieve selectivity.

**Figure 4:**
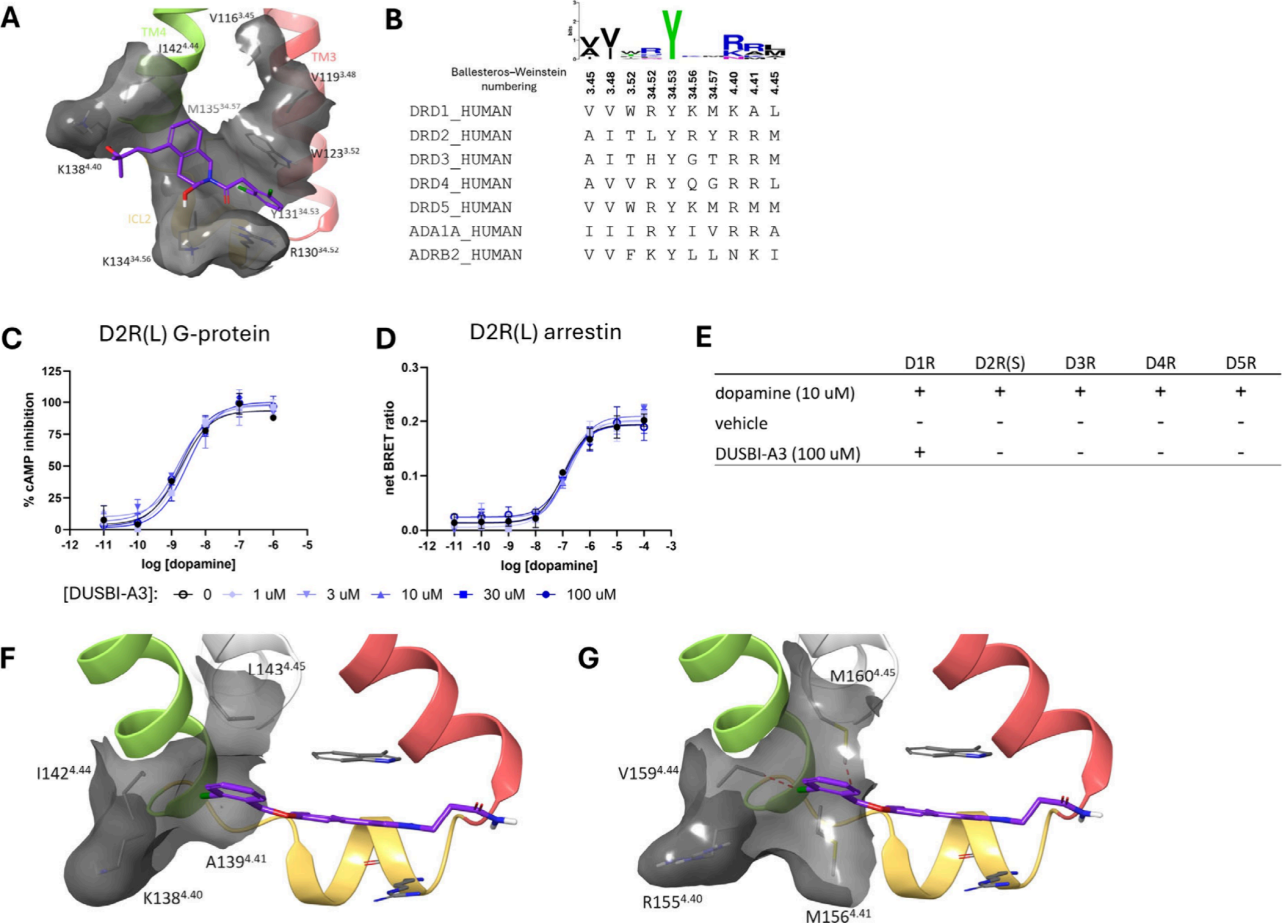
Sequence analysis and alignment at the ICL2 allosteric pocket and selectivity screening of DUSBI-A3. (A) Structural analysis of 10 residues that constitute the ICL2 allosteric pocket in the hD1R. The receptor surface is shown in gray, with TM2, ICL2, TM3, and LY3154207 highlighted in red, yellow, green, and violet, respectively. (B) Sequence alignment of these 10 residues across all human dopamine receptors and human α1 and β2 adrenergic receptors. (C, D) Dose–response curves for dopamine activating the G protein pathway (C) or the β-arrestin pathway (D) in the absence or presence of DUSBI-A3 at the D2R­(L). (E) β-Arrestin-mediated receptor translocation studies in cells overexpressing different dopamine receptors treated with vehicle, DUSBI-A3 (100 μM), or dopamine (10 μM). (F, G) ICL2 allosteric pocket of D1R (F) and a D5R model (G). The receptor surface is shown in gray with TM2, ICL2, TM3, and LY3154207 highlighted in red, yellow, green, and violet, respectively. Red lines indicate steric clashes.

To assess receptor selectivity among various dopaminergic receptors, we tested DUSBI-A3’s ability to affect dopamine-induced cAMP inhibition assays and β-arrestin recruitment assays at the long isoform of the dopamine D2 receptor (D2R­(L)). The results suggested that DUSBI-A3 did not affect dopamine induced D2R signaling with concentrations up to 100 μM (Figure 4C,D). Additionally, we used confocal microscopy to follow receptor translocation after treatment with the vehicle, the β-arrestin-biased allosteric modulator DUSBI-A3, or dopamine. Our results show DUSBI-A3 induced receptor translocation in cells overexpressing hD1R, but not in cells expressing other dopamine receptors (Figure 4E, Supplementary Figure 1). To identify potential differences between D1R and D5R that may lead to DUSBI-A3’s preference to D1R over D5R, we started with our docking pose and virtually mutated four key residues in the pocket to corresponding amino acids in D5R. The results suggest that while A139, I142 and L143 form a pocket that surrounds the benzene chlorobenzene ring on DUSBI-A3 (Figure 4F), I142 V and L143 M mutations lead to steric clashes with DUSBI-A3 that may prohibit its binding (Figure 4G). These findings confirm that allosteric ligands targeting the ICL2 allosteric pocket can achieve receptor selectivity, likely due to the sequence diversity within this site.

## Discussion

Development of receptor-selective and signaling-biased ligands has remained a challenge, despite an impressive array of conventional high throughput technological approaches developed over the past few decades. Since this resource-intensive approach failed for us in establishing a biased hit D1R lead antiparkinsonian candidate, we leveraged newly acquired cryo-EM structures to enable virtual D1R computer screening. Utilizing the ZINC compound database and an expanding pool of commercially available compounds,^42^ we completed a virtual screen of 4 times the number of machine screened compounds in just 4 days using a standard desktop computer. The identified hit compounds were acquired from commercial vendors at an average cost of $55 per compound over a time frame of weeks. Following the initial screening round, we resynthesized only a limited number of bioactive compounds for validation, substantially minimizing what is typically a demanding synthetic workload. The streamlined approach in which virtual screening becomes the initial step of the discovery process greatly facilitates and expedites novel drug development using minimal resources. This approach is particularly well-suited for orphan targets, small laboratories, limited budgets, and abbreviated timelines.

Our study emphasizes the importance of choosing an appropriate model for virtual screening. In the search for allosteric modulators, we began with two distinct structures of the D1R. By docking known ligands, we presumably captured a structure that more accurately represents the likely interactions of an allosteric ligand, and this work also highlights the role of conformational changes involving position W123 of the ICL2 in the D1R. Additional molecular dynamics (MD) calculations can validate whether each pose is energetically stable, whereas free energy perturbation (FEP) calculations can also contribute to this analysis. Thus, we believe our discovery of novel D1R allosteric ligands was only successful due to the rational selection of model (PDB: 7LJC) over (PDB: 7CKZ).

The D1R remains a prominent drug target for PD.^10^ Notably, we demonstrated that the ICL2 allosteric pocket is an efficient target for achieving receptor specificity. Through amino acid sequence alignment with closely related GPCRs, we identified diversity in the ICL2 allosteric pocket that supports receptor-selective ligands. Using confocal microscopy, compound DUSBI-A3 was identified as selectively activating arrestin-mediated receptor translocation at D1R but not at related GPCRs. Similarly, allosteric ligands recognized for targeting the β2 adrenergic receptor (β2AR) at the ICL2 pocket have shown strong selectivity over β1AR.^43^ In contrast, the orthosteric pockets of different dopamine receptors are remarkably similar, presenting significant challenges for developing selective orthosteric ligands.^11^

Biased allosteric modulators are an emerging solution to the unmet need of reduced off-target therapeutic signaling.^21,25^ Moreover, mutations in the ICL2 region of various GPCRs have been shown to generate mutant receptors biased toward either G protein or arrestin pathways.^44–46^ A key finding from our study that should be emphasized is the general potential for the ICL2 allosteric pocket of GPCRs to serve as a site model for developing signaling-biased ligands. Importantly, the ICL2 interacts directly with both G proteins^39^ and β-arrestins,^47^ supporting a potential role in biased signaling. In addition, the D1R DRY motif, residues, D^3.49^, R^3.50^, and Y^3.51^ that play a critical role in activation,^48^ are adjacent to key functional residues W^3.52^ and Y^3.53^. Moreover, the Y^34.53^ on the ICL2, which was predicted as part of the ICL2 pocket and to directly interact with allosteric ligands, was shown to participate in an ionic lock motif with D^3.49^ and R^3.50^ in the D3R, the β1AR and the A_2A_R.^49^ Notably, our virtual screening that sought compounds potentially binding to the ICL2 pocket yielded biased ligands, further demonstrating the ICL2 allosteric pocket as a hotspot for the development of biased allosteric ligands.

We discovered two arrestin biased allosteric modulators at D1R, which are interesting hits for further developments. The two ligands are predicted to bind to the same pocket in the ICL2 with very similar poses for the D1R, while sharing no chemical similarity among themselves and with known allosteric ligands for the site, thus, highlighting the power of virtual screening to identify new ligands with diverse scaffolds. These two ligands are allosteric and can be used with L-DOPA to selectively enhance its arrestin activity at D1R, thereby minimizing side effects from other receptors. The compounds demonstrated promising activities at as low as 10 μM and signaling bias toward the β-arrestin pathway by simultaneously inhibiting the G-protein pathway and enhancing the β-arrestin pathway by dopamine. Between them, DUSBI-C5 appears to be the more robust in favoring the arrestin pathway in bioassays but weaker under confocal microscopy assay. The molecule also has challenges in chemistry, with two chiral centers and five steps in synthesis. In comparison, DUSBI-A3 has no chiral center, can be synthesized in three steps, and demonstrates stronger arrestin-mediated receptor translocation under the microscope. In conclusion, we foresee DUSBI-A3 to be the superior hit for further medicinal chemistry development.

In summary, small druggable molecules that bind to the D1R ICL2 allosteric pocket can induce conformational changes, resulting in signaling bias. As such, the ICL2 allosteric pocket is a promising area for developing ligands with a virtual screening platform. This approach applies not only to the D1R and its application to PD, but also, in general, to the larger rhodopsin class of therapeutic GPCRs.

## Materials and Methods

### Cell Culture and Transfections

Human embryonic kidney HEK-293T cells were obtained from the American Type Culture Collection (Manassas, VA). The U2OS cell lines stably expressing the target receptor and GFP-tagged β-arrestin-2 were generated by our laboratory.^18,19^ All cells were maintained in DMEM (Gibco, Grand Island, NY) supplemented with 10% fetal bovine serum (FBS, Sigma-Aldrich, St. Louis, MO) and 1% antibiotic antimycotic solution (A5955, Sigma-Aldrich) in a humidified atmosphere at 37 °C with 5% CO_2_. Transfections were conducted using a standard calcium phosphate method.^50^

### Molecular Docking

Molecular docking was performed using the Maestro platform (Schrodinger, New York, NY). Structures of the hD1R with PDB codes 7LJC, 7CKZ, and 7JVQ were obtained from the Protein Data Bank. Heterotrimeric G proteins were removed, and the receptor–ligand complexes were prepared using the Protein Preparation Wizard. Receptor grids were generated through the Glide Receptor Grid Generation function and centered on the allosteric site, with the grid dimensions approximating the binding pocket of LY3154207 from the corresponding resolved structures. Ligands for docking were generated and processed using the LigPrep function with the OPLS_2005 force field. Docking simulations were then performed using Glide’s Ligand Docking module, with the receptor grid and preprocessed ligands as inputs. The docking protocol employed flexible ligand sampling in the standard precision (SP) mode.

### Virtual Screening

A data set of 2.5 million 3-D compound structures was sourced from the ZINC database, filtered to include compounds with a molecular weight between 350 and 500, log *P* values of 3–5, and designated as “in-stock” for commercial availability. A random sample of approximately 500000 compounds was selected as a starter set. These structures were then screened virtually by docking into the receptor grid, using the Glide’s high-throughput virtual screening (HTVS) mode. Compounds with a docking score of −5.1 or better were retained, narrowing the pool to approximately 11000 compounds. These compounds were next processed using LigPrep to maintain stereochemistry while generating potential ionization states within the pH range of 6–8. The refined set of ligands underwent a second round of virtual screening in the SP mode. Applying a docking score threshold of −5.6, the top 2011 compounds were clustered using the Tanimoto coefficient (0.3 threshold) in RDKit, yielding 200 clusters. The highest-scoring compound from each cluster was manually reviewed for its stability, toxicity, and pharmacokinetic profile. Finally, 26 promising compounds in their racemic mixture form were ordered from MolPort (Latvia) for functional assay testing.

### GloSensor cAMP Accumulation Assay

HEK-293T cells were seeded into 6-well plates at 7.5 × 10^5^ cells per well and were cotransfected with 200 ng of hD1R and 3 μg of GloSensor-22F (Promega, Madison, WI) using calcium phosphate transfection. Twenty-four h post-transfection, cells were plated into clear-bottom, white-walled 96-well plates pretreated with poly-d-lysine at 50,000 cells/well in “BRET media” - clear minimum essential medium (Gibco) supplemented with 2% FBS, 10 mM HEPES, 1× GlutaMax, and 1× antibiotic antimycotic solution (Sigma-Aldrich). The following day, media were removed, and cells were incubated at room temperature with 25 μL of HHBSS buffer (Hanks’ balanced salt solution (Gibco) supplemented with 20 mM HEPES) containing 8 μM d-luciferin for 2 h. Twenty-five μL of HHBSS buffer was added to each well, and cells were treated with 40 μL of either vehicle (HHBSS alone) or the indicated concentration of test drug for 5 min. Cells were stimulated with 10 μL of dopamine at the indicated final concentrations. Luminescence was measured using a microplate reader (CLARIOstar, BMG Labtech, Cary, NC) 10 min after dopamine stimulation. Dose–response curves were generated with GraphPad Prism 9.

### BRET β-Arrestin-2 Recruitment Assay

HEK-293T cells were seeded into 6-well plates at 7.5 × 10^5^ cells per well and were cotransfected with 100 ng of hD1R-Rluc8, 1.5 μg of Venus-β-arrestin-2 and 1 μg pcDNA3.1 using calcium phosphate transfection. Twenty-four h post transfection, cells were plated into clear bottom, white-walled 96-well plates pretreated with poly-d-lysine at 50000 cells/well in “BRET media” - clear minimum essential medium (Gibco) supplemented with 2% FBS, 10 mM HEPES, 1× GlutaMax, and 1× antibiotic antimycotic solution (Sigma). The following day, media were removed, and cells were incubated at room temperature with 50 μL of HHBSS buffer (Gibco) containing 3 μM coelenterazine-H (NanoLight) for 15 min. Cells were treated with 40 μL of either vehicle (HHBSS alone) or the indicated concentration of the test drug for 5 min. Cells were then stimulated with 10 μL of dopamine at the indicated final concentrations. Luminescence at 475–30 nm (donor) and 535–40 nm (acceptor) were monitored with a microplate reader (CLARIOstar, BMG Labtech) 10 min after treatment. BRET ratios were calculated as acceptor signals divided by donor signals. Dose–response curves were generated with GraphPad Prism 9.

### Confocal Receptor Translocation Assay

The confocal receptor translocation assay has been previously described.^23^ U2OS cells stably expressing β-arrestin-2-GFP and various receptors were seeded into black, optical-bottom 96-well plates (Nunc) at a density of 20000 cells per well in Opti-MEM supplemented with 2% FBS. After 24 h, the media were removed and replaced with 60 μL of Opti-MEM. Cells were then treated with either 40 μL of vehicle (Opti-MEM) or the indicated compound and incubated in a humidified chamber (5% CO_2_, 37 °C) for 40 min. Treatment was stopped by adding 33 μL of 4% paraformaldehyde and incubating for 20 min at room temperature. Fixed cells were either stored at 4 °C or immediately imaged for β-arrestin-2-GFP translocation using a Zeiss LSM 510 Meta confocal microscope.

### Synthesis of hD1R Biased Allosteric Modulators

Synthesis of the hD1R biased allosteric modulators DUSBI-A3 and DUSBI-C5 is described in detail in the Supporting Information. ^1^H NMR spectra were recorded using a 400 MHz spectrometer with compounds dissolved in DMSO. HPLC analyses were performed with two methods. Method 1: Analysis was performed on a Shimadzu Prominence-I LC-2030C 3D Plus (Shimadzu, Japan) instrument. A 5.5 min gradient of 5% to 95% acetonitrile in water (containing 0.05% ammonium hydroxide) was used at a flow rate of 1.5 mL/min. A Waters X-bridge BEH C_18_ column (3 μm, 3 mm × 30 mm) was used at a temperature of 45 °C. Method 2: Analysis was performed on a Shimadzu LCMS-2020 (Shimadzu, Japan) instrument. A 1.8 min gradient of 5% to 95% acetonitrile in water (containing 0.05% ammonium hydroxide) was used with a 3 min run-time at a flow rate of 1.0 mL/min. A Waters X-bridge C_18_ column (3.5 μm, 2.1 mm × 50 mm) was used at a temperature of 50 °C. The chiral purity of compounds was analyzed using supercritical fluid chromatography (SFC) on a SHIMADZU LC-30AD system. The method employed was IB N-5-40%D-2.5, using a DAICEL IB N-5 column (4.6 mm × 250 mm, 5 μm particle size). The mobile phase consisted of a mixture of CO_2_ and methanol (MeOH) in a 60:40 ratio with 0.1% ammonia (7 M in MeOH) as an additive. The flow rate was set to 2.5 mL/min, and the column oven was maintained at 40 °C throughout the analysis. Purity was determined based on HPLC chromatograms using both Method 1 and Method 2, and SFC analysis. All compounds were at least 95% purity. Mass determination was performed using a Shimadzu LCMS-2020 with electrospray ionization in the positive mode. The ^1^H NMR spectra, HPLC chromatograms, SFC chromatograms and mass spectra are found in the Supporting Information.

## Supplementary Material



## Data Availability

The docking poses of the hit compounds docked to D1R structure are in PDB format, the CSV files containing molecular descriptors and a Python script for categorizing virtual screening hit compounds according to their Tanimoto similarity coefficient are available at GitHub repository (https://github.com/Barak-Group/D1R-allosteric-virtual-screening.git).

## Notes

Compound synthesis information on DUSBI-A3 and DUSBI-C5, including synthetic schemes, synthetic procedures, ^1^H NMR spectra, HPLC chromatograms, mass spectrum, SFC analysis. Images for confocal receptor translocation studies (PDF)

## ABBREVIATIONS

ADHD: attention-deficit hyperactivity disorder
BRET: bioluminescence resonance energy transfer
cAMP: cyclic adenosine monophosphate
D1R: dopamine D1 receptor
FDA: Food and Drug Administration
GPCR: G protein coupled receptor
GFP: green fluorescent protein
HPLC: high-performance liquid chromatography
HTVS: high-throughput virtual screening
ICL2: intracellular loop 2
LCMS: liquid chromatography–mass spectrometry
L-DOPA: levodopa
MeOH: methanol
NMR: nuclear magnetic resonance
PD: Parkinson’s disease
PDB: Protein Data Bank
SFC: supercritical fluid chromatography
TM: transmembrane
