# Supplementary material for: Virtual Compound Screening for Discovery of Dopamine D1 Receptor Biased Allosteric Modulators
Source: J Chem Inf Model. 2025 Sep 11;65(18):9713–22. doi: 10.1021/acs.jcim.5c00972 (PMC12478865; doi:10.1021/acs.jcim.5c00972)
Supplement: Supplementary file 1 [file ci5c00972_si_001.pdf]

Supporting Information

Virtual Compound Screening for Discovery of Dopamine D1 Receptor Biased Allosteric Modulators

Yang Zhou[a], William Wetsel[b,c], Steven H. Olson[d,], and Lawrence S. Barak[a,*]*

[a]Department of Cell Biology, Duke University Medical Center, Durham, NC 27710;

[b]Departments of Psychiatry and Behavioral Sciences, Cell Biology, and Neurobiology, Duke University Medical Center, Durham, NC 27710, USA;

[c]Mouse Behavioral and Neuroendocrine Analysis Core Facility, Duke University Medical Center, Durham, NC 27710, USA

[d]Conrad Prebys Center for Chemical Genomics at Sanford Burnham Prebys Medical Discovery Institute, La Jolla, CA 92037, USA;

*To whom correspondence may be addressed. Email: solson@sbpdiscovery.org or lawrence.barak@duke.edu.

Table of Contents

Synthesis of DUSBI-C5……………………………………………………...S1
Synthetic scheme……………………………………………………...S1
Synthetic procedures…………………………………………………S1
^{1}H NMR spectra……………………………………………………S4
HPLC chromatograms………………………………………………...S4
Mass spectrum………………………………………………………...S5
SFC analysis…………………………………………………………..S6
Synthesis of DUSBI-A3…………………………...……………………....S7
Synthetic scheme……………………………………………………...S7
Synthetic procedures…………………………………………………S7
^{1}H NMR spectra…………………………………………….…………S9
HPLC chromatograms……………………………………….………..S9
Mass spectrum………………………………………………………S10
Supplementary Figure 1……………………………………………………S11

Synthesis of DUSBI-C5

Synthetic Scheme of DUSBI-C5

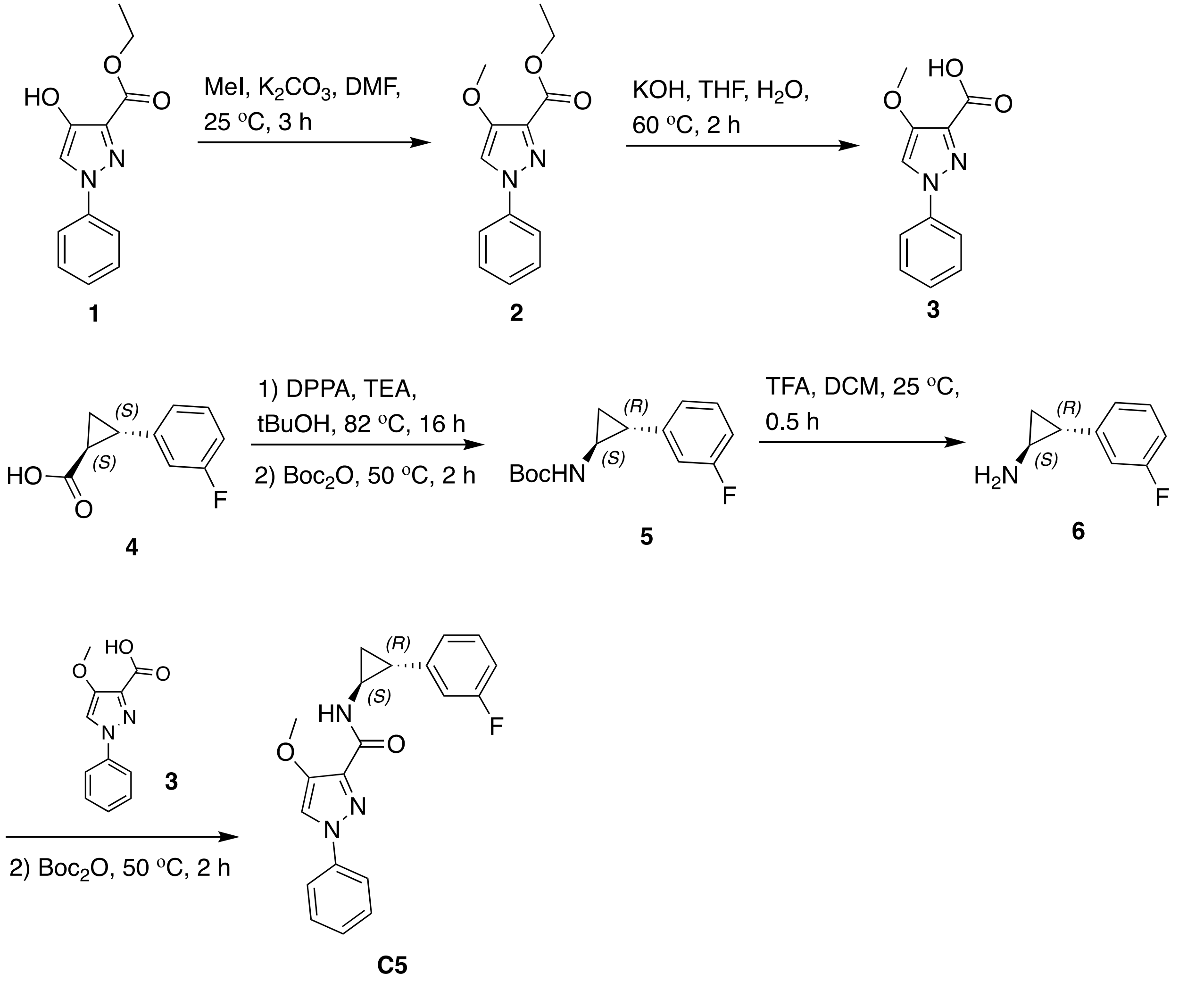

<u>Synthetic procedure:</u>

<u>Step 1: ethyl 4-methoxy-1-phenyl-1H-pyrazole-3-carboxylate (2)</u>

A mixture of ethyl 4-hydroxy-1-phenylpyrazole-3-carboxylate (**1**, 1 g, 4.30 mmol), MeI (0.79 g, 5.60 mmol) and K_2CO_3 (0.77 g, 5.60 mmol) in DMF (15 mL) was stirred at 25 °C for 3 hours. After the reaction was completed, the mixture was diluted with water (20 mL), the pH was adjusted to 7-8 with saturated $NaHCO_3$ and extracted with EtOAc (10 mL x 3). The combined organic layers were dried over Na_2SO_4, filtered and concentrated under reduced pressure to

obtain a residue. The residue was purified by flash chromatography column (eluent EtOAc in petroleum ether = 53%) to give **Cpd. 3** (0.6 g, 51% yield) as an orange oil.

MS (ESI): mass calcd. for $C_{13}H_{14}N_2O_3$ 246.10 m/z found 246.8.

^{1}H NMR (400 MHz, CDCl3-d_6): δ 7.73 – 7.71 (m, 2H), 7.60 (s, 1H), 7.46 (t, J = 8.0 Hz, 2H), 7.33 (t, J = 7.6 Hz, 1H), 4.46 (q, J = 7.2 Hz, 2H), 3.92 (s, 3H), 1.43 (t, J = 7.2 Hz, 3H).

Step 2：4-methoxy-1-phenyl-1H-pyrazole-3-carboxylic acid (3)

A mixture of ethyl 4-methoxy-1-phenylpyrazole-3-carboxylate (**2**, 600 mg, 2.44 mmol) and KOH (410 mg, 7.31 mmol) in THF/H_2O (10 mL/10 mL) was stirred and heated at 60 ^oC for 2 hours. After the reaction was completed, the mixture was diluted with water (10 mL), the pH was adjusted to 1-2 with 1 M HCL and extracted with EtOAc (10 mL x 3). The organic layer was dried over Na_2SO_4, filtered and concentrated to obtain **Cpd. 3** (400 mg, 68% yield) as a white solid.

MS (ESI): mass calcd. for $C_{11}H_{10}N_2O_3$ 218.07 m/z found 218.8.

^{1}H NMR (400 MHz, CDCl3-d_6): δ7.73 – 7.71 (m, 2H), 7.65 (s, 1H), 7.51 – 7.47 (m, 2H), 7.39 – 7.35 (m, 1H), 3.97 (s, 3H).

Step 3：tert-butyl ((1*S*,2*R*)-2-(3-fluorophenyl)cyclopropyl)carbamate (5)

To a solution of (1*S*,2*S*)-2-(3-fluorophenyl)cyclopropane-1-carboxylic acid (**4**, 680 mg, 3.77 mmol) in toluene (12 mL) was added DPPA (1246 mg, 4.53 mmol), TEA (649 mg, 6.42 mmol) and t-BuOH (6154 mg, 83.03 mmol). The mixture was stirred and heated at 82 ^oC for 16 hours. Then the mixture was cooled to 50 ℃, followed by the addition of Boc_2O (1236 mg, 5.66 mmol). The resulting mixture was stirred and heated at 50 ℃ for another 2 hours. After the reaction was completed, the mixture was diluted with water (10 mL) and extracted with EtOAc (10 mL x 3). The combined organic layers were dried over Na_2SO_4, filtered and concentrated under reduced pressure to obtain a residue. The residue was purified by flash chromatography column (eluent EtOAc in petroleum ether = 9%) to give **Cpd. 5** (120 mg, 11% yield) as a white solid.

MS (ESI): mass calcd. for $C_{14}H_{18}FNO_2$ 251.13 m/z found 250.90 $[M-H]^-$.

^{1}H NMR (400 MHz, CDCl3-d_6): δ 7.24 – 7.18 (m, 1H), 6.92 (d, J = 7.6 Hz, 1H), 6.88 – 6.80 (m, 2H), 4.82 (s, 1H), 2.72 – 2.71 (m, 1H), 2.06 – 2.01 (m, 1H), 1.45 (s, 9H), 1.18 – 1.15 (m, 2H).

Step 4: (1*S*,2*R*)-2-(3-fluorophenyl)cyclopropan-1-amine (6)

To a solution of tert-butyl *N*-[(1*S*,2*R*)-2-(3-fluorophenyl)cyclopropyl]carbamate (**5**, 120 mg, 0.48 mmol) in DCM (3 mL) was added TFA (3 mL). The mixture was stirred at 25 °C for 0.5 hours. After the reaction was completed, the mixture was concentrated to obtain **Cpd. 6** (80 mg, 68% yield) as a yellow oil.

MS (ESI): mass calcd. for $C_9H_{10}FN$ 151.08, m/z found 151.8 $[M+H]^+$.

^1H NMR (400 MHz, $CDCl_3$) δ 7.25 (s, 1H), 6.96 – 6.91 (m, 1H), 6.85 (d, *J* = 7.6 Hz, 1H), 6.76 (d, *J* = 9.6 Hz, 1H), 2.84 – 2.83 (m, 1H), 2.50 – 2.49 (m, 1H), 1.33 – 1.26 (m, 2H).

Step 5: N-((1R,2S)-2-(3-fluorophenyl)cyclopropyl)-4-methoxy-1-phenyl-1H-pyrazole-3-carboxamide (DUSBI-C5)

A mixture of (1*S*,2*R*)-2-(3-fluorophenyl)cyclopropan-1-amine (**6**, 80 mg, 0.53 mmol), 4-methoxy-1-phenylpyrazole-3-carboxylic acid (**3**, 115 mg, 0.53 mmol), TCFH (594 mg, 2.12 mmol) and NMI (261 mg, 3.18 mmol) in MeCN (5 mL) was stirred at 25 °C for 2 hours. After the reaction was completed, the mixture was diluted with water (5 mL) and extracted with EtOAc (5 mL x 3). The combined organic layers were dried over Na_2SO_4, filtered and concentrated under reduced pressure to obtain a residue. The residue was purified by flash chromatography column (eluent EtOAc in petroleum ether = 50%) to give the crude as a yellow oil. The crude was purified by reversed phase chromatography column (mobile phase : [H_2O (0.05% FA-H_2O)-ACN]; B%: 45% - 63%, 20 min) to obtain the racemic compound as a white solid. The compound was separated by prep-SFC (column:Daicel Chiralpak IH-10 SFC; mobile phase: A: CO_2; B:MeOH [0.1%NH_3 (7 M solution in MeOH)]= 60/40) to obtain **DUSBI-C5** (22.55 mg, 11 % yield) as a white solid.

MS (ESI): mass calcd. for $C_{20}H_{18}FN_3O_2$ 351.14, m/z found 352.2 $[M+H]^+$.

^1H NMR (400MHz, MeOD): *δ* 8.17 (s, 1H), 7.85 – 7.83 (m, 2H), 7.53 – 7.46 (t, *J* = 8.0 Hz, 2H), 7.36 – 7.32 (m, 1H), 7.30 – 7.25 (m, 1H), 7.03 (d, *J* = 7.6 Hz, 1H), 6.98 – 6.95 (m, 1H), 6.92 – 6.87 (m, 1H), 3.93 (s, 3H), 3.05 – 3.01 (m, 1H), 2.24 – 2.19 (m, 1H), 1.42 – 1.28 (m, 2H).

^{19}F NMR (377MHz, MeOD): -116.04.

NMR spectrum:

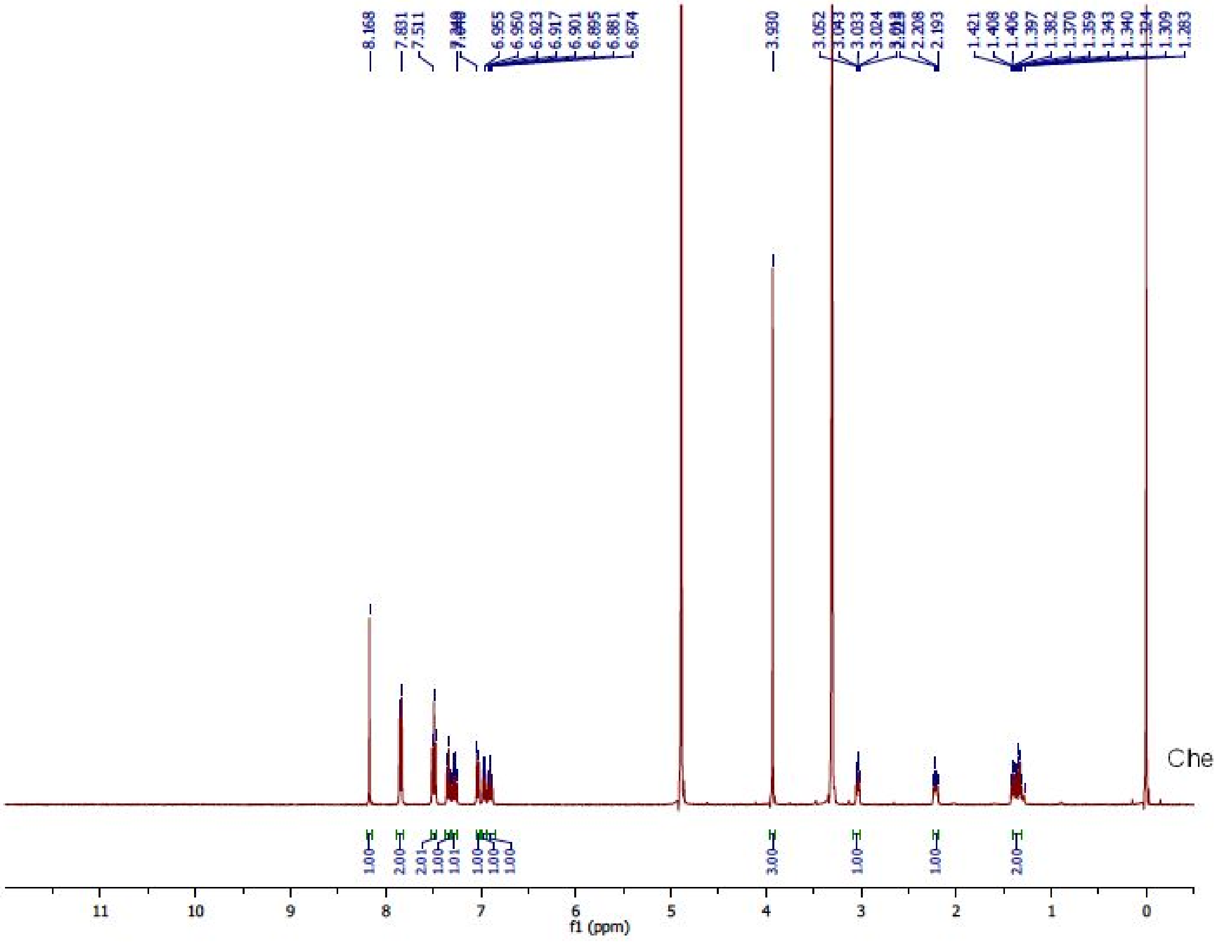

HPLC chromatogram system 1:

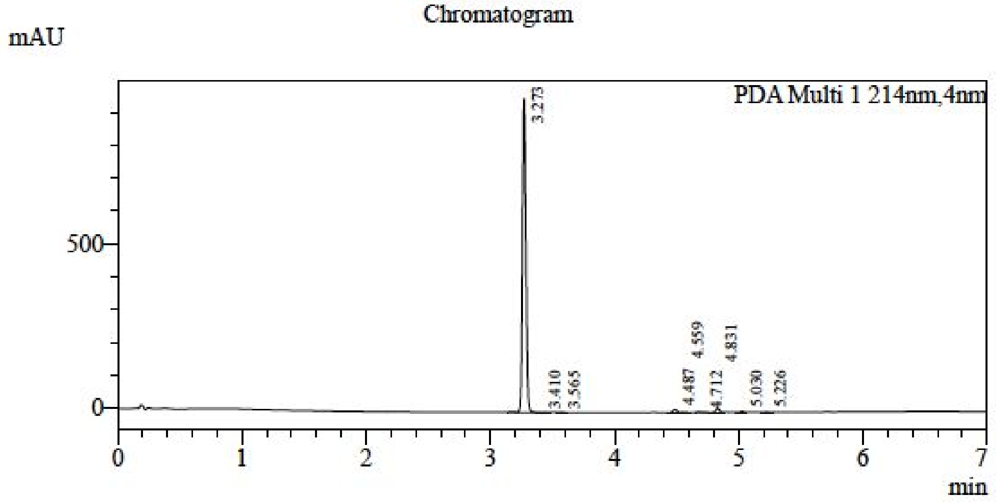

HPLC chromatogram system 2:

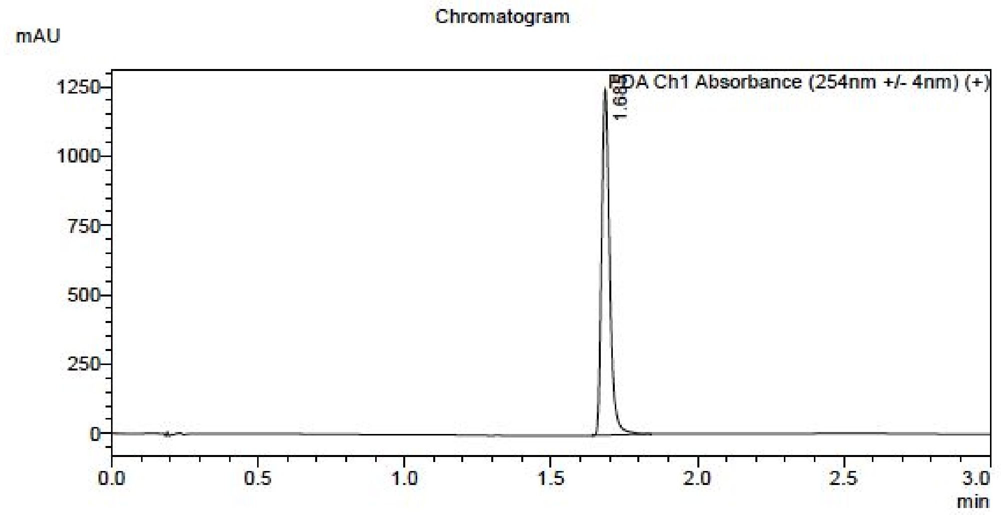

Mass spectrum:

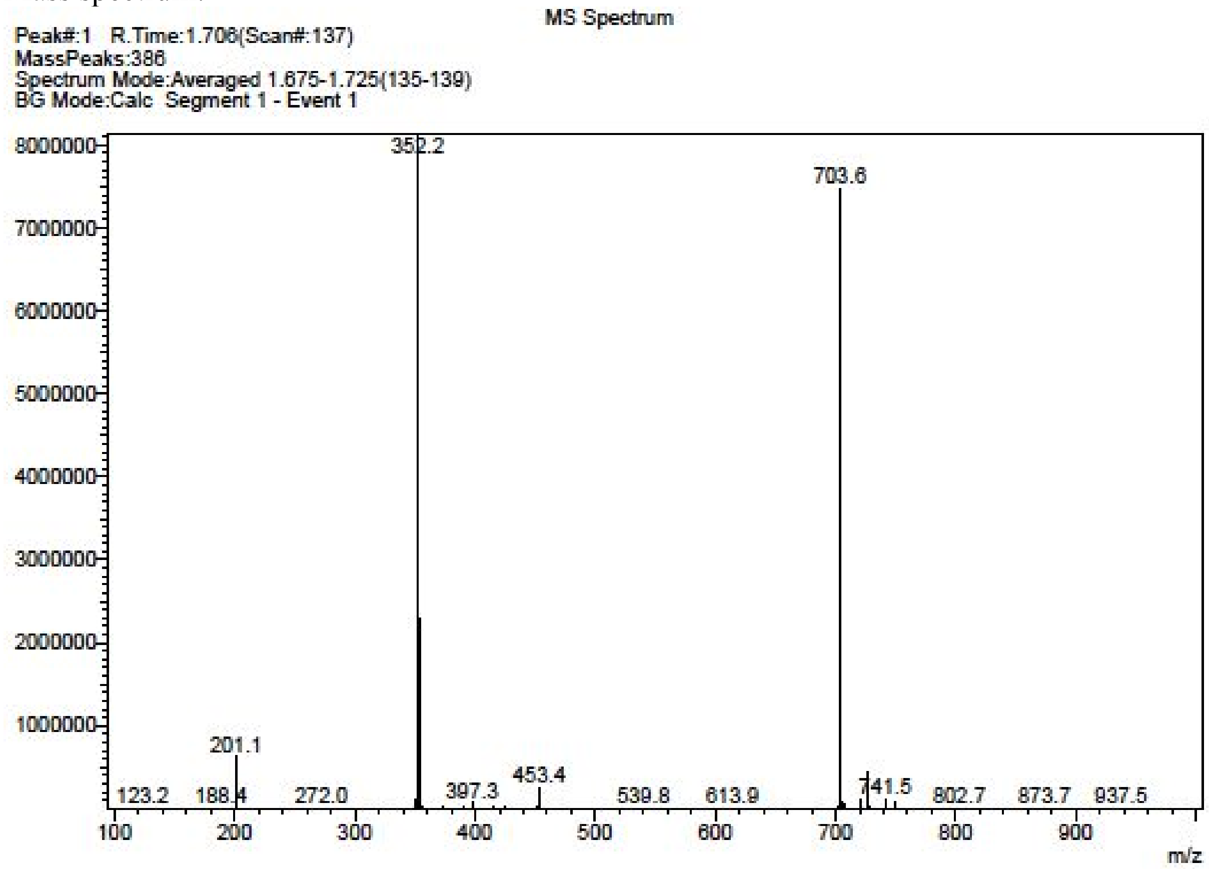

SFC analysis:

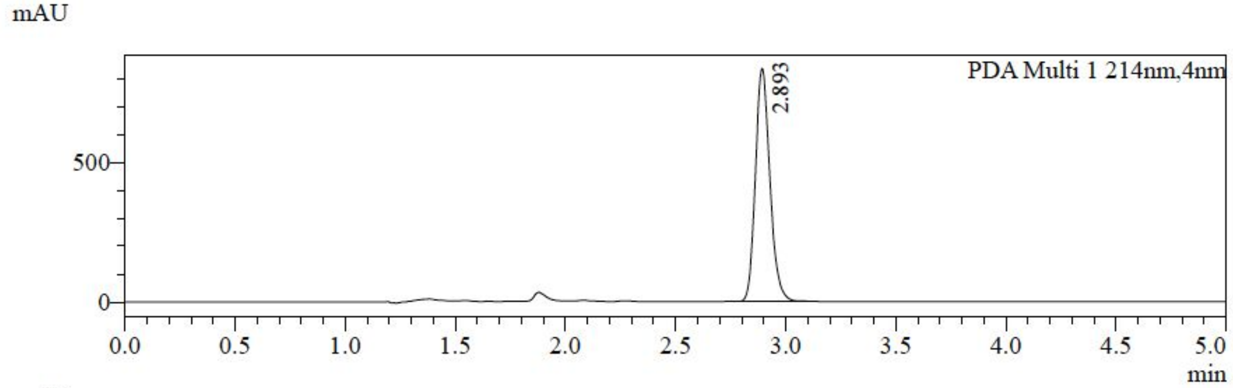

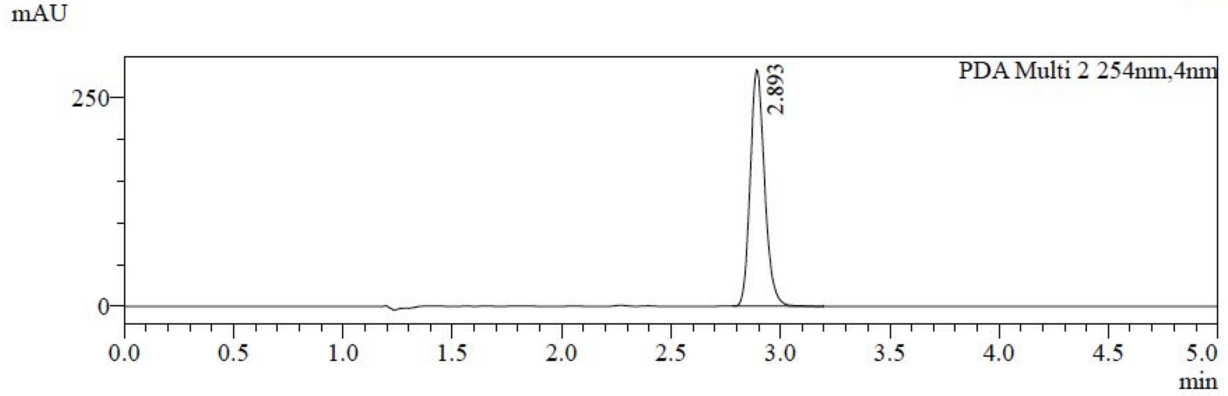

Synthesis of DUSBI-A3

Synthetic Scheme of DUSBI-A3

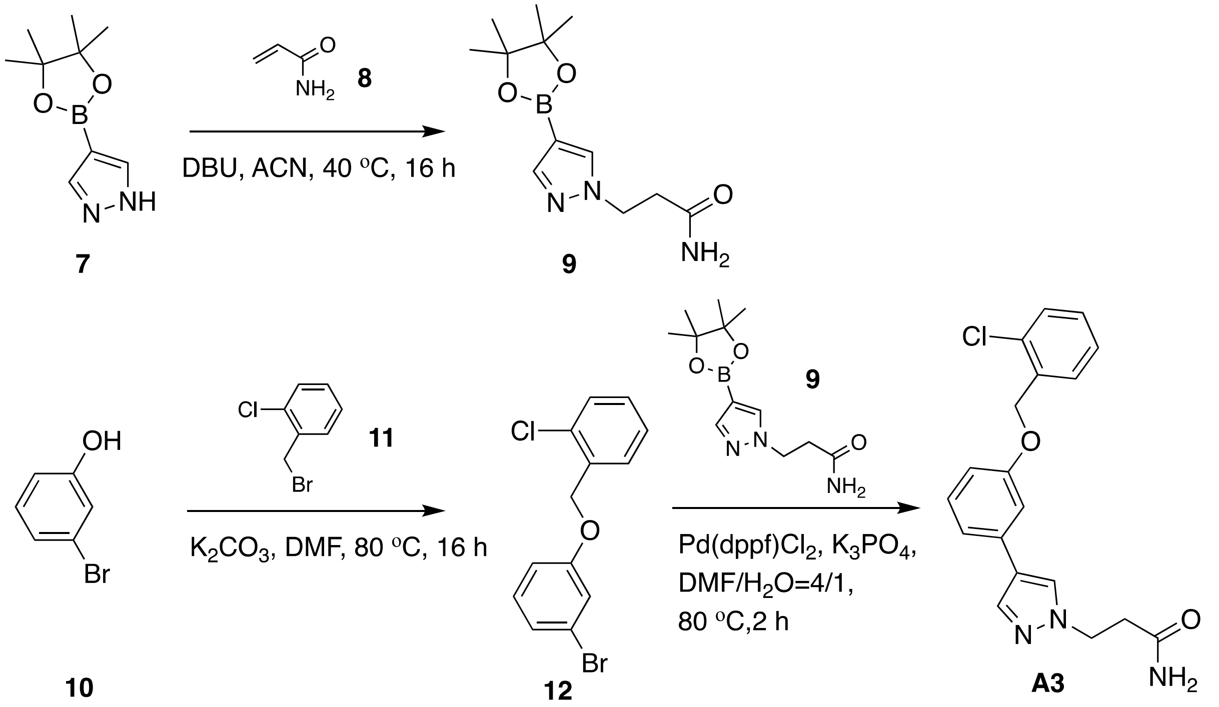

Synthetic procedure:

Step 1：3-(4-(4,4,5,5-tetramethyl-1,3,2-dioxaborolan-2-yl)-1H-pyrazol-1-yl)propanamide (9)

A mixture of 4-(4,4,5,5-tetramethyl-1,3,2-dioxaborolan-2-yl)-1H-pyrazole (**7**, 1.0 g, 5.20 mmol), prop-2-enamide (**8**, 0.74 g, 10.40 mmol) and DBU (0.40 g, 2.60 mol) in MeCN (10 mL) was stirred at 40 °C for 16 hours. The mixture was diluted with water (20 mL) and extracted with EtOAc (10 mL x 3). The combined organic layers were dried over Na_2SO_4, filtered and concentrated under reduced pressure to obtain a residue. The residue was purified by flash chromatography column (eluent with MeOH in DCM = 20%) to give the **Cpd. 9** (1 g, 65% yield) as a colorless oil.

MS (ESI): mass calcd. for $C_{12}H_{20}BN_3O$ 265.16, m/z found 265.8 $[M+H]^+$.

^1H NMR (400MHz, $CDCl_3$): δ 7.84 (s, 1H), 7.57 (s, 1H), 7.38 (s, 1H), 6.89 (s, 1H), 4.30 (t, J = 6.8 Hz, 2H), 2.61 (t, J = 6.8 Hz, 2H), 1.24 (s, 12H).

Step 2：1-((3-bromophenoxy)methyl)-2-chlorobenzene (12)

A mixture of 3-bromophenol (**10**, 350 mg, 2.02 mmol), 1-(bromomethyl)-2-chlorobenzene (**11**, 499 mg, 2.43 mmol) and K_2CO_3 (336 mg, 2.43 mmol) in DMF (5 mL) was stirred and heated at 80 ^oC for 16 hours. The mixture was diluted with water (20 mL) and extracted with EtOAc (10 mL x 3). The combined organic layers were dried over Na_2SO_4, filtered and concentrated under reduced pressure to obtain a residue. The residue was purified by flash chromatography column (eluent with EtOAc in petroleum ether = 1-2%) to give the **Cpd. 12** (0.35 g, 52% yield) as a white solid.

MS (ESI): mass calcd. for $C_{13}H_{10}BrClO$ 295.96, m/z found 280.0 $[M+H]^+$.

^1H NMR (400MHz, $CDCl_3$): δ 7.54 – 7.52 (m, 1H), 7.42 – 7.39 (m, 1H), 7.32 – 7.27 (m, 2H), 7.18 – 7.10 (m, 3H), 6.93 – 6.90 (m, 1H), 5.14 (s, 2H).

Step 3: 3-(4-(3-((2-chlorobenzyl)oxy)phenyl)-1H-pyrazol-1-yl)propenamide (DUSBI-A3)

A mixture of 1-(3-bromophenoxymethyl)-2-chlorobenzene (**12**, 150 mg, 0.50 mmol), 3-[4-(4,4,5,5-tetramethyl-1,3,2-dioxaborolan-2-yl)pyrazol-1-yl]propanamide (**9**, 134 mg, 0.50 mmol), Pd (pddf)Cl_2*DCM (41 mg, 0.05 mmol) and K_3PO_4 (321 mg, 1.51 mmol) in DMF (8 mL) and H_2O (2 mL) was stirred and heated at 80 ^oC for 2 hours. The reaction mixture was diluted with water (10 mL) and extracted with EtOAc (10 mL x 3). The combined organic layers were dried over Na_2SO_4, filtered and concentrated under reduced pressure to obtain a residue. The residue was purified by flash chromatography column (eluent with MeOH in DCM = 17%) to give the **DUSBI-A3** (80.61 mg, 43% yield) as a brown solid.

MS (ESI): mass calcd. for $C_{19}H_{18}ClN_3O_2$ 355.11, m/z found 355.9 $[M+H]^+$.

^1H NMR (400MHz, DMSO-d_6): δ 8.14 (s, 1H), 7.89 (s, 1H), 7.65 – 7.62 (m, 1H), 7.55 – 7.51 (m, 1H), 7.41 – 7.39 (m, 3H), 7.29 – 7.24 (m, 2H), 7.16 (d, J = 7.6 Hz, 1H), 6.91 (s, 1H), 6.86 – 6.83 (m, 1H), 5.19 (s, 2H), 4.30 (t, J = 6.8 Hz, 2H), 2.65 (t, J = 6.8 Hz, 2H).

NMR spectrum

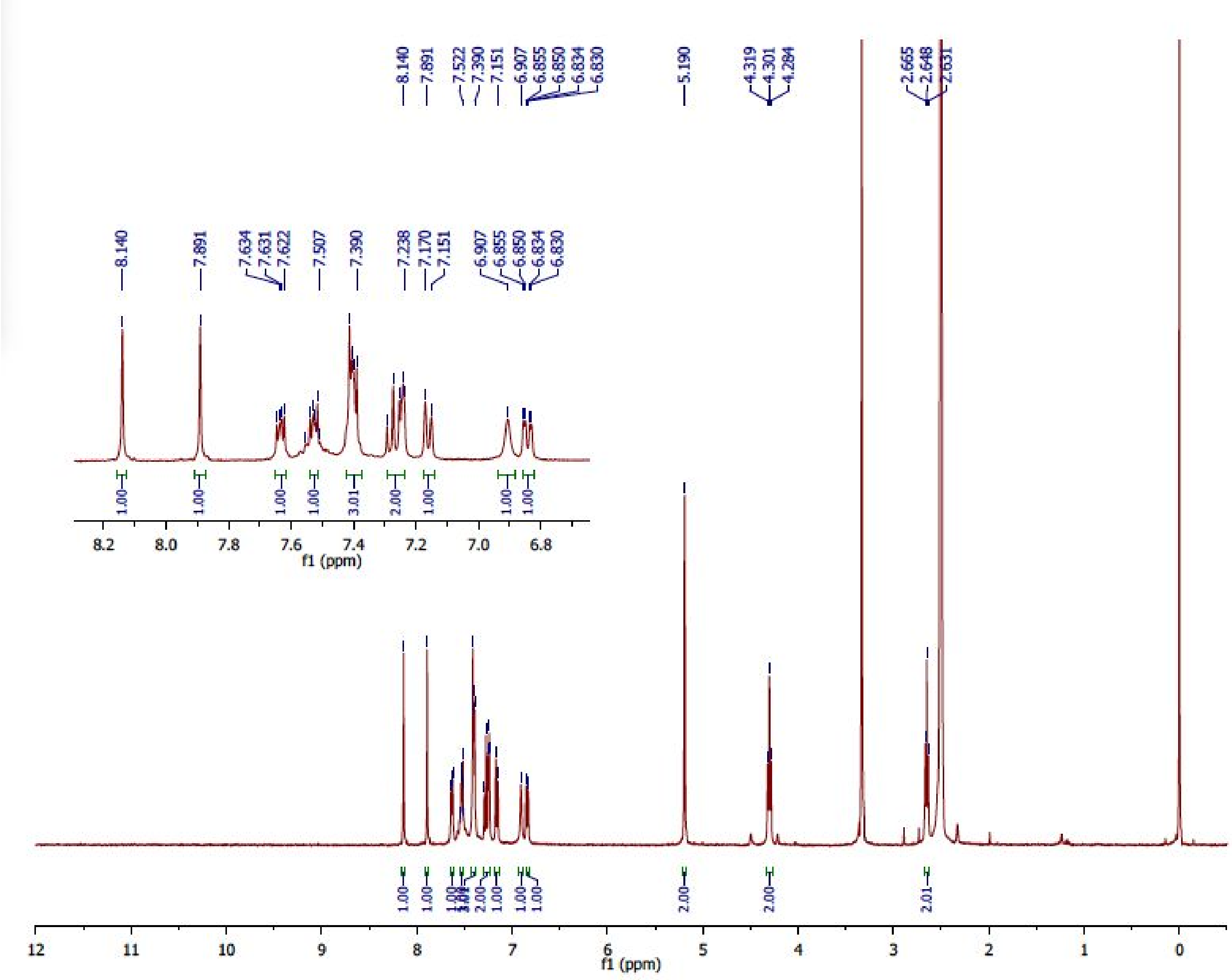

HPLC chromatogram system 1:

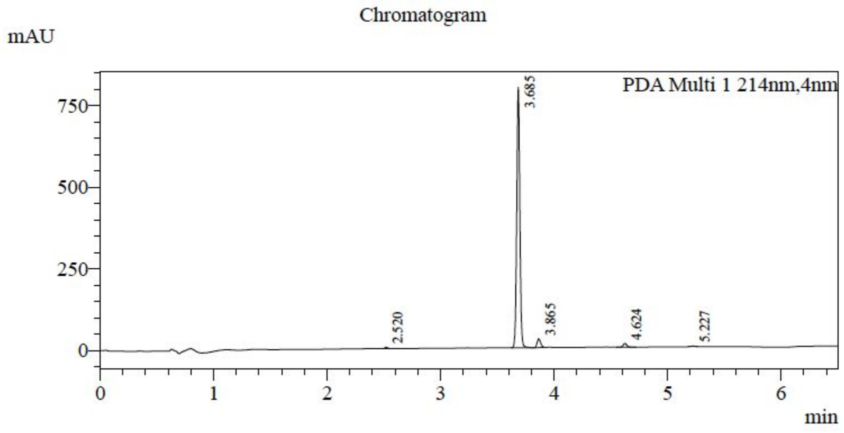

HPLC chromatogram system 2:

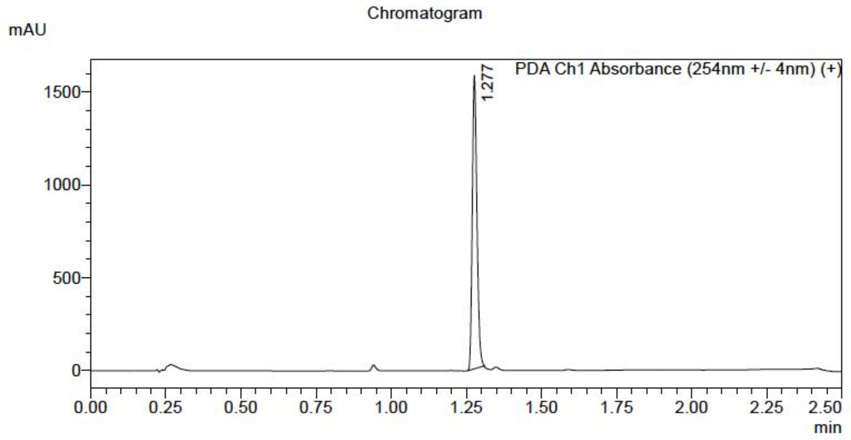

Mass spectrum:

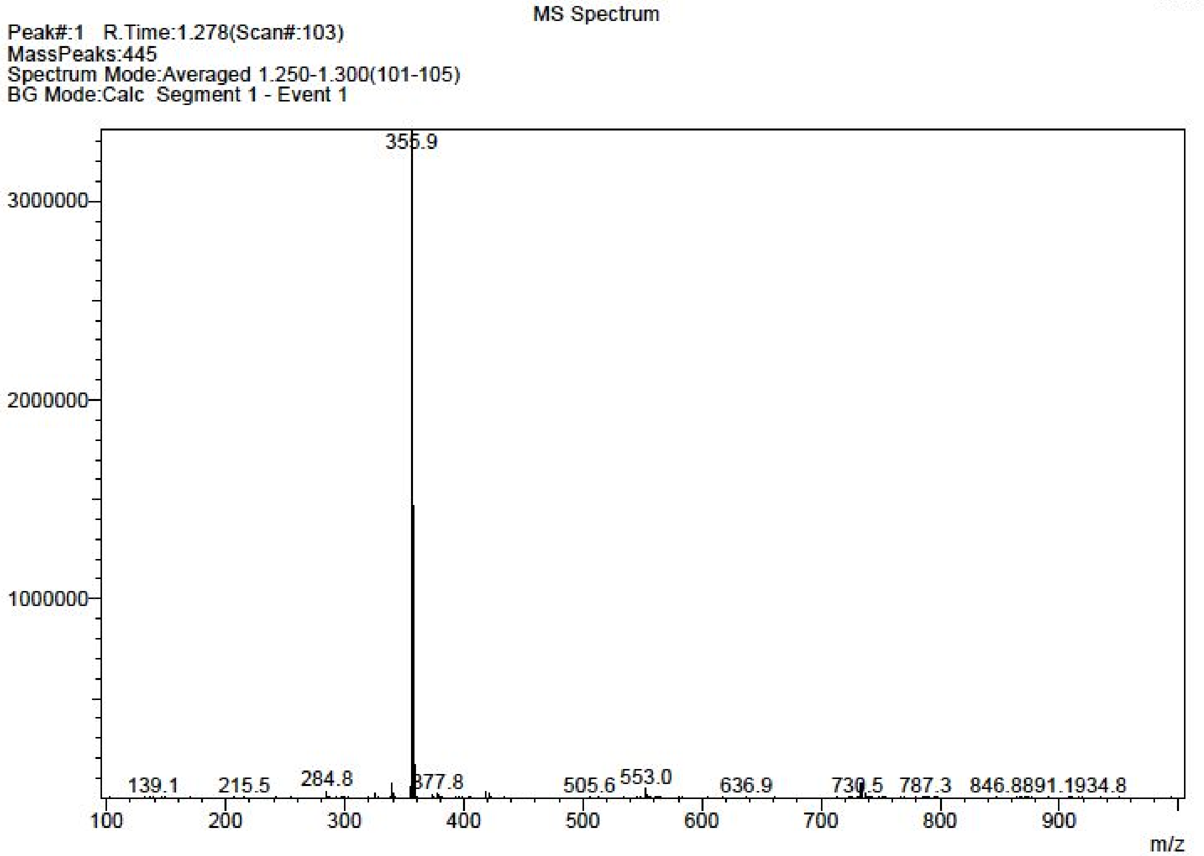

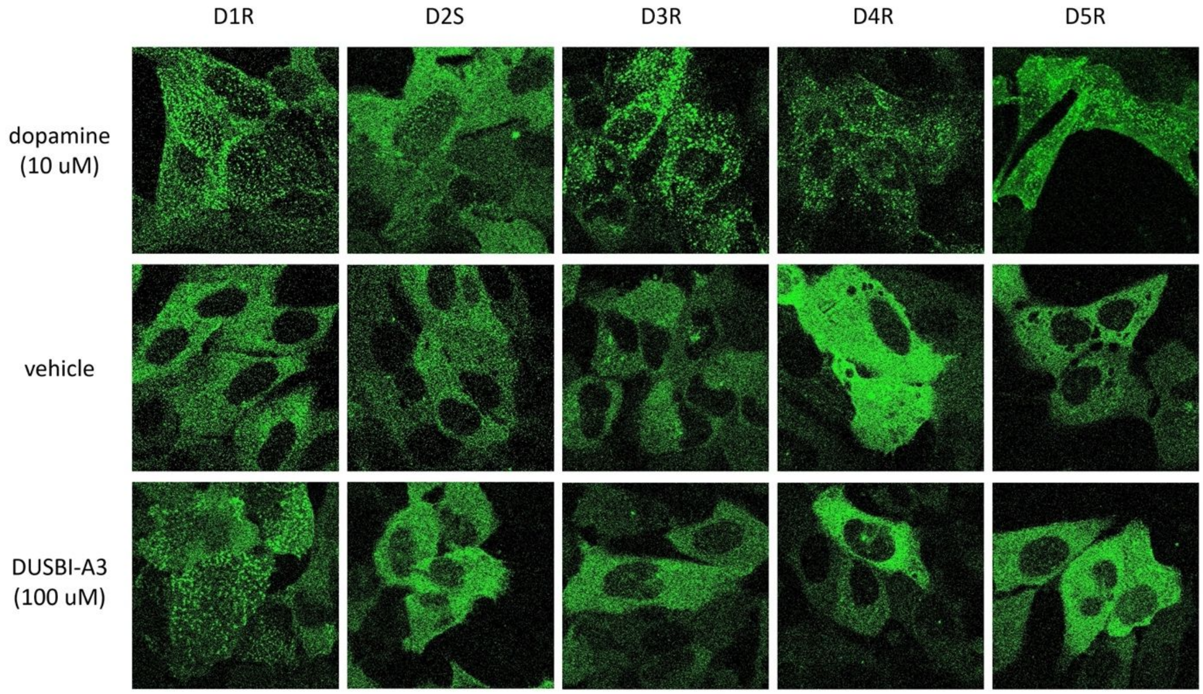

Supplementary Figure 1. β-arrestin mediated hD1R receptor translocation studies with vehicle, DUSBI-A3 (100 μM) or dopamine (10 μM).
